# Letter to the Editor: Prevalence and cardiovascular associations of elevated lipoprotein(a) in people living with HIV: A cross-sectional study

**DOI:** 10.1016/j.clinme.2026.100647

**Published:** 2026-09-23

**Authors:** Ellesa Ahmad Nor Hisham, Devon Ward

**Affiliations:** University Hospitals of Liverpool Group, United Kingdom; University Hospitals of Liverpool Group, United Kingdom; University of Liverpool, United Kingdom

Dear Editor,

We read with interest the paper exploring cardiovascular associations of elevated lipoprotein(a) (Lp(a)) in people living with HIV (PLHIV).^1^ We highlight to readers the importance of ethnicity when interpreting these conclusions.

As the authors acknowledge, Lp(a) concentrations vary across ethnic groups. Prior multiethnic studies demonstrated a genetically determined association with elevated Lp(a),^2^ more specifically that Black participants have significantly higher Lp(a) concentrations than White participants.^3^ This is important to Lago *et al*.’s study given the marked imbalance in ethnic representation: only three of the 186 participants being Black compared to 107 White participants. In the UK, HIV positivity rates are five times higher among Black than White patients.^4^ This is important as the authors draw conclusions regarding the prevalence of PLHIV, in a sample population that is not representative of the PLHIV population, with such a small proportion of Black participants represented.

We argue that the four mutually exclusive ethnic group categories in Lago *et al*.’s study are not sufficient when investigating Lp(a) in PLHIV. Self-reported Hispanic/Latino ethnicity does not capture genetic variation in this category. If Lp(a) and cardiovascular associations are influenced by genetic background as research suggests, findings from a predominantly White and Hispanic/Latino cohort may not be generalisable to other PLHIV populations.

While ethnicity was included in Lago *et al*.’s multivariable model as an important determinant, there were too few Black and Asian participants for this adjustment to be informative. Though the underrepresentation of these ethnic groups was recognised, the impact of this limitation on their conclusion regarding the relative contribution of genetic and HIV-related factors warrants greater emphasis.

In conclusion, Lago *et al*.’s study provides valuable insight into the relationship between elevated Lp(a) and cardiovascular associations in PLHIV, an under-represented population in Lp(a) research. However, genetic background, notably in Black and Asian cohorts, is associated with higher Lp(a). We therefore question the conclusion that the sample size was adequate and encourage future studies to include broader ethnicity categories and adequate representation of ethnic groups.

## CRediT authorship contribution statement

**Devon Ward:** Conceptualization, Writing – original draft, Writing – review & editing. **Ellesa Ahmad Nor Hisham:** Conceptualization, Writing – original draft, Writing – review & editing.

## Funding

This research did not receive any specific grant from funding agencies in the public, commercial, or not-for-profit sectors.

## Declaration of competing interest

The authors declare that they have no known competing financial interests or personal relationships that could have appeared to influence the work reported in this paper.
